# Children transition from simple associations to explicitly reasoned social learning strategies between age four and eight

**DOI:** 10.1038/s41598-022-09092-1

**Published:** 2022-03-23

**Authors:** Kirsten H. Blakey, Elizabeth Renner, Mark Atkinson, Eva Rafetseder, Christine A. Caldwell

**Affiliations:** 1 Psychology, Faculty of Natural Sciences, University of Stirling, Stirling, UK; 2 Philosophy, Faculty of Arts and Humanities, University of Stirling, Stirling, UK; 3 Department of Psychology, Durham University, Durham, UK; 4 School of Management and School of Psychology and Neuroscience, University of St Andrews, St Andrews, UK

**Keywords:** Psychology, Human behaviour, Cultural evolution

## Abstract

To differentiate the use of simple associations from use of explicitly reasoned selective social learning, we can look for age-related changes in children’s behaviour that might signify a switch from one social learning strategy to the other. We presented 4- to 8-year-old children visiting a zoo in Scotland (*N* = 109) with a task in which the perceptual access of two informants was determined by the differing opacity of two screens of similar visual appearance during a hiding event. Initially success could be achieved by forming an association or inferring a rule based on salient visual (but causally irrelevant) cues. However, following a switch in the scenario, success required explicit reasoning about informants’ potential to provide valuable information based on their perceptual access. Following the switch, older children were more likely to select a knowledgeable informant. This suggests that some younger children who succeeded in the pre-switch trials had inferred rules or formed associations based on superficial, yet salient, visual cues, whereas older children made the link between perceptual access and the potential to inform. This late development and apparent cognitive challenge are consistent with proposals that such capacities are linked to the distinctiveness of human cumulative culture.

## Introduction

The ability to focus social learning on knowledgeable others has been proposed as a cognitive capacity underpinning distinctively human cumulative culture: the accumulation of beneficial modifications to cultural traits over successive generations of learners resulting in increased functionality or efficiency^1–4^. To some extent targeted learning which focusses on those who possess relevant knowledge or experience has been observed in both humans and non-human animals (henceforth animals) in the form of social learning strategies (SLSs)^5–10^. However, there are marked differences between humans and animals with regards to the capacity for cumulative culture to account for^2,11^. While recent evidence highlights some capacity for cumulative culture in animals^12–14^, its expression appears to be context-specific and relatively restricted compared to human adults^3,15,16^.

The distinctiveness of human cumulative culture has recently been attributed to the capacity to use *explicitly metacognitive SLSs*^17–19^. These are rules which are consciously represented and (for verbal individuals) reportable, that reflect an explicit awareness and understanding of the learning strategies being employed. Learners who employ an explicitly metacognitive SLS have an appreciation of why their strategy is successful because they understand the causal link between another’s knowledge or experience and their value as a source of information. This type of SLS is thought to enable tracking of *when* others are likely to have superior knowledge, and *who* of the available others is likely to be the best source of knowledge, so that social learning can be most efficient^17,18,20^. For example, when uncertain about a boat-building problem, copy the boat builder with the largest fleet^21^. As humans appear to be the only animals that focus their social learning by asking ‘who knows’, this targeted learning towards appropriate social sources is believed to, at least in part, facilitate cumulative culture^22,23^. The ability to take others’ knowledge and experience into account may at the very least broaden the contexts in which cumulative culture is likely to occur^17^.

However, the majority of the behaviours that conform to SLSs in both humans and animals likely arise as a consequence of general-purpose associative learning processes or biologically selected biases^18^. In contrast to explicitly metacognitive SLSs, they are not driven by causal understanding of the potential value of social information, rather, these *implicit SLSs* are based on relatively crude heuristics. Implicit SLSs—and selective learning more broadly—direct learning towards objects, agents, and events that are most likely to provide useful information^6,7^; examples include ‘copy older individuals’ and ‘copy the majority’. Though referred to as implicit, we do not claim that learners are necessarily unaware of personal preferences that guide their social learning. It is likely that learners sometimes explicitly represent strategies related to salient, yet superficial, cues without appreciating the causal relationship between the cue and success. Such explicit strategies are not explicitly metacognitive due to the absence of a causal understanding of the relevance of the informants’ mental states.

Many researchers claim that from as early as 3 to 4 years old children’s selective social learning is the result of sophisticated reasoning about the epistemic competence of others^24,25^, while explanations involving associative accounts reserved for only early forms of selective social learning^26–28^. Some of the evidence from the SLSs^9,29^ and *selective trust*^30–32^ literature suggests that very young children^33–36^ (between 3 and 4 years old) and animals^8,37,38^ (e.g., chimpanzees and capuchin monkeys) preferentially learn from knowledgeable individuals (e.g., biases for copying older or more successful others and trusting previously accurate over inaccurate informants). However, these biases are not necessarily driven by explicitly metacognitive SLSs. Indeed, much of the current empirical evidence of SLSs in animals and young children could be explained equally well in terms of associative learning processes^18,21,39,40^. For example, biases for older, high-status, or reliable models may be the result of repeated exposure to the successes of models with these characteristics (either established as part of the experimental procedure or from personal experience), thus resulting in learned associations or rule-like strategies (implicit SLSs)^19^. Implicit SLSs are, by their nature, effective heuristics, and therefore likely to generate a similar behavioural outcome as a more cognitively demanding explicitly metacognitive strategy, making it difficult to justify claims involving higher-order mechanisms. In the current study, to reveal whether implicit or explicitly metacognitive SLSs are being employed in a given situation, we set these in conflict with one another.

Some researchers suggest that SLSs may be culturally learned, so it should be noted that much of the developmental research we discuss (including the current study) has been conducted in WEIRD societies (Western, Educated, Industrialised, Rich, and Democratic^41^), therefore we should be cautious about making generalisations^42^. We focus our investigation on 4- to 8-year-old children, a population expected to encompass both those that rely more heavily on implicit strategies, and those more likely to employ explicitly metacognitive reasoning. Human adults are—justifiably—assumed to be able to use explicitly metacognitive SLSs when seeking out and using social information^18,43,44^. Explicitly metacognitive SLSs, and their prerequisite cognitive capacities, are likely to be experience-dependent and learned through social interaction^18^. As such, they likely emerge relatively late in development after children have had the opportunity to learn through experience. Motivations for seeking out appropriate social models are proposed to be based on advanced metacognitive abilities^18,45^. Thus, the cognitive challenge posed by such abilities may preclude young children (and animals) from identifying the most appropriate source of social information. Therefore, we would not expect to see explicit understanding of the value of social information, and how one might benefit from it, in infants or very young children (under 5 years)^46^. It may be possible to differentiate the SLSs that children can use by identifying age-related changes in social information use that might signify a switch from implicit to explicitly metacognitive strategies. Determining whether age-related changes coincide with advances in cognitive development could offer insight into the prerequisite mechanisms and help to explain why cumulative culture appears to be restricted in animals.

Recent evidence indicates an age-related transition from the use of implicit to explicitly metacognitive SLSs that, consistent with the above account, occurs relatively late in childhood (from around 6–7 years)^19^. Though they did not need to reason about others’ mental states, older children (7–8 years) were found to direct their learning towards others’ who possessed relevant information, consistent with an ability to use explicitly metacognitive SLSs^19^. By contrast, younger children (3–6 years) selected informants (incorrectly) based on model-based biases for gender congruence. In the current study we sought to examine whether children can identify an appropriate source of information based on a causal understanding of the link between the others’ knowledge or experience and their value as a source of information (i.e., tracking who knows). Thus, it was necessary to offer a scenario in which the appropriate choice could not be predicted without consideration of others’ knowledge states.

Children appear to be able to use others’ perceptual experience to determine their knowledge or ignorance from 3 to 4 years old^47–51^. Several studies have used ‘perspective-taking’ paradigms to investigate whether non-human primates^52,53^ or young children^54^ understand the relationship between seeing and knowing. Heyes^55^ claimed that in many such studies, preference for a knowledgeable informant could be explained by formation of an associative rule across trials, and proposed a novel methodology. Heyes’s proposal involved giving an individual first-hand experience of a novel barrier that granted or denied perceptual access, before testing them to see whether they project similar mental states onto others who face the same barrier. In such a case, to identify the perceptual access afforded to others individuals must use personal experience^56^. Accordingly, preference for an agent whose barrier permits perceptual access would suggest attribution of knowledge states based on self-experience of the barrier’s properties, as opposed to a response reinforced by prior experience (e.g., preference for head orientation).

**Figure 1:**
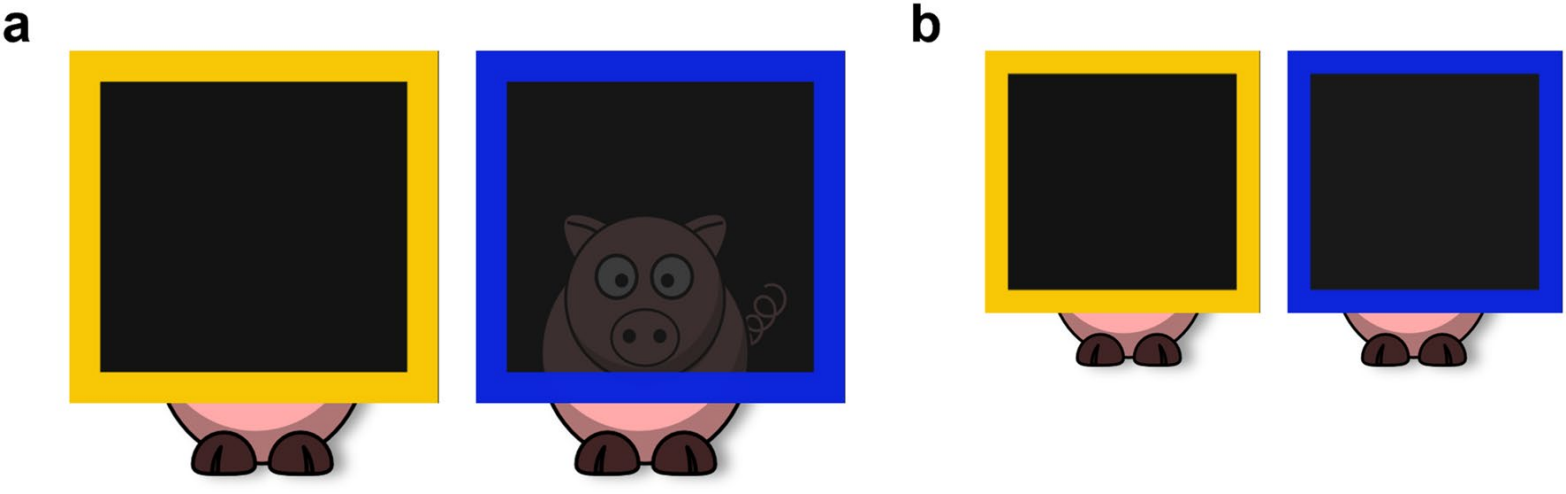
Illustration of the perceptual access afforded by the opaque (yellow) and semi-opaque (blue) screens when interrupting the view of another object. (**a**) when viewed close up, illustrative of child’s view during the experience phase; (**b**) when viewed at a distance, illustrative of child’s view during pre-switch and switch phases.

In this study the perceptual access of two informants was determined by the differing opacity of two screens of similar visual appearance (i.e., degree of transparency—opaque or semi-opaque—could only be determined by looking directly through each one at close range, see Fig. 1). This meant that children could not use prior knowledge or existing associations, rather, they had to learn new rules related to novel task cues. This was important for determining children’s capacity to attribute mental states based on a causal understanding of access to desirable information. To differentiate implicit and explicitly metacognitive SLSs, we created a situation in which it was possible to initially succeed using associative learning or inferred rules (i.e., implicit SLSs), without the need for causally relevant insights into such processes.

The task comprised three sequential phases. An *experience phase* provided participants with information about the perceptual access afforded by the screens. A *pre-switch phase* presented a scenario in which participants could form an association, or infer a rule, between one of a number of potentially salient visual cues and the desired outcome which did not require explicit reasoning of informants’ perceptual access to a critical event. In each trial, before hiding the reward, the semi-opaque screen was placed in front of one informant (knowledgeable) and the opaque screen was placed in front of the other (ignorant). Participants were required to select which informant would tell them where the reward was hidden. Reminiscent of animal cognition paradigms^57–60^, participants were exposed to repeated pre-switch trials until they reached a proficiency criterion (or completed 10 trials), at which point they progressed to the next phase. Reaching criterion was taken to mean that children had formed an association or inferred a rule that predicted success, or that they had understood the causal link between informants’ perceptual access and their knowledge. Finally, a *switch phase* induced a switch in the task scenario (see Method section) following which success required an understanding of the causal link between informants’ perceptual access and their knowledge. The switch generated a conflict between the response favoured by associations, or inferred rules, that predicted success in the pre-switch phase and the response favoured by an understanding of why another’s behaviour is informative. To explore whether participants were truly responding to the informants’ perceptual access or if they had simply made the connection between the reward and screen (or the colour of its frame); perceptual access was indicated by the informants facing, and facing away from, the hiding event in the final two switch trials.

Both the pre-switch and switch phases measured success as selection of the knowledgeable informant. We were particularly interested in what the children who reached the proficiency criterion had learned during the pre-switch trials. We examined their responses in the switch phase to establish whether they had inferred a causal link involving perceptual access, or had formed another non-mentalistic association, or inferred a rule, based on superficial cues. Older children were expected to be more likely to reason about the value of the informants’ knowledge based on their perceptual access to the critical event (explicitly metacognitive SLSs). Whereas we expected that younger children would rely on simple associations, or inferred rules, learned during the pre-switch trials (implicit SLSs). If children were using explicitly metacognitive SLSs, we would expect them to reach criterion and continue to be successful in the switch trials, while lower success in the switch trials (despite reaching criterion) could indicate that children were instead relying on implicit SLSs. To assess explicit understanding more directly we also asked children to justify their choice of informants. Recent evidence shows that by 5 years, children can give explicit, valid justifications in their reasoning and decision-making^61,62^.We anticipated that verbal responses would shed light on what was driving children’s choices, as well as potentially offering support for the interpretation of behavioural responses.

## Results

The analysis assessed the switch phase performance of children who met the proficiency criterion. Analyses were performed using R^63^, with generalised linear mixed effects analyses (GLMMs) performed using *lme4*^64^ with logit regression and ordinal regressions performed using the *ordinal* package^65^. *P* values < 0.05 were accepted as statistically significant. The binary dependent variable in all GLMMs was the selection of the knowledgeable informant in the switch trials. Age was centred and scaled to measure thousands of days. Where specified as fixed effects, the following variables were sum coded: met criterion (Criterion not met as − 1, Criterion met as 1), presence of screens (No screens as − 1, Screens as 1) and number of trials required to meet criterion (> 5 trials as − 1, 5 trials as 1). The random effects structure for each model aimed to include by-participant random slopes for all fixed effects and keep random effects structures ‘maximal’ where possible^66^. Where necessary, random slopes were removed, followed by random intercepts, until a convergent, non-singular model was obtained. To check for collinearity between predictor variables Variance Inflation Factors (VIFs) were calculated for standard linear models excluding random effects and interactions using the *performance* package^67^. Collinearity was not an issue in any of the models (VIFs ≤ 1.03).

### Switch phase performance

#### Effect of reaching the proficiency criterion

**Figure 2:**
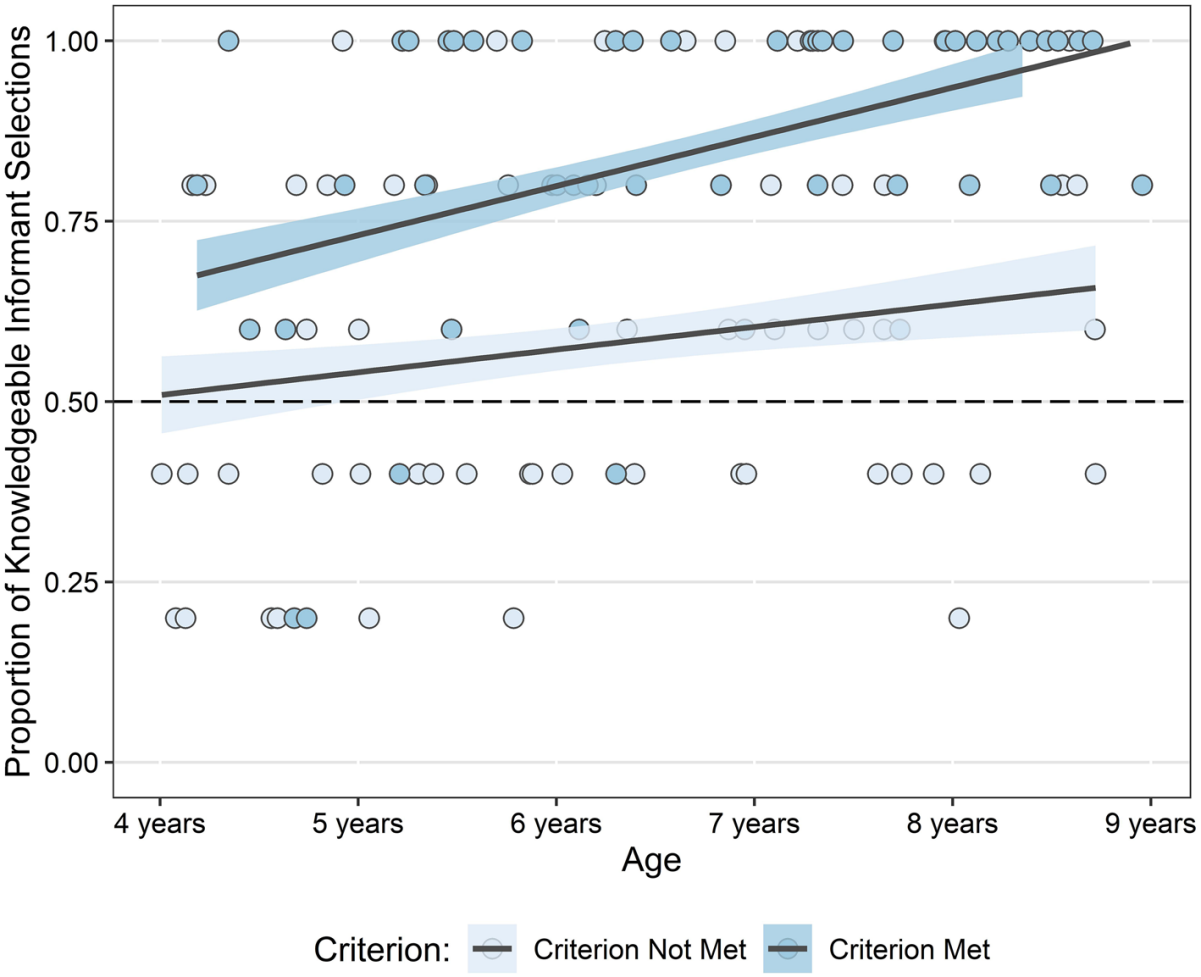
Proportion of knowledgeable informant selections in the switch trials by age and whether children met criterion or not. Age is plotted by age in days. Dashed line indicates chance performance.

Overall, 52 children (48%) reached the proficiency criterion (Table 1). To examine the effect of reaching the proficiency criterion on performance we built a GLMM for selection of the knowledgeable informant in each switch trial with fixed effects of age, met criterion, the presence of screens, and all interactions among these variables, with random intercepts of trial number and participant ID. This model was significantly better than the null equivalent (*χ*^2^(7) = 48.8, *p* < 0.001). Significant main effects of age (*p* < 0.001) and meeting criterion (*p* < 0.001) revealed that older children and children who met criterion selected the knowledgeable informant more often than younger children and those who did not meet criterion. A significant two-way interaction between age and meeting criterion (*p* = 0.033) showed that the effect of meeting criterion was greater in older children (Fig. 2). There was no evidence of a main effect nor any interactions involving the presence of screens (*p* ≥ 0.544). For full model output see Supplementary Table S1.

**Table 1:** Number of children who met criterion by age in years (*N* = 109) and, for those who met criterion (*N* = 52), the number of pre-switch trials required to do so.

Age	*n*	Criterion met		No. of pre-switch trials required to meet criterion	
		No	Yes	5 trials	> 5 trials
4 years	21	14	7 (33%)	3	4
5 years	23	13	10 (43%)	6	4
6 years	22	11	11 (50%)	8	3
7 years	23	11	12 (52%)	8	4
8 years	20	8	12 (60%)	9	3
All	109	57	52 (48%)	34	18

The total number of knowledgeable informant selections participants made in the switch phase was compared to chance (50%) using one-sample *t*-tests. Children who met criterion selected the knowledgeable informant in an average of 4.23 switch trials; significantly above chance *t*(51) = 11.78, *p* < 0.001, two-tailed. Children who did not meet criterion also selected the knowledgeable informant significantly above chance in 2.89 switch trials *t*(56) = 2.36, *p* = 0.022, two-tailed. An independent-samples *t-*test showed that the difference between these two groups was significant *t*(106.27) = 6.00, *p* < 0.001, two-tailed.

#### Age-effects in children who met the proficiency criterion

To address whether age affected the likelihood of using explicit metacognitive strategies to determine the appropriate informant, we constructed a GLMM for selection of the knowledgeable informant in each switch trial for children who met criterion. The GLMM had fixed effects of age, the presence of screens, and the interaction between these variables, with a random intercept of participant ID. The model was significantly better than its null equivalent (*χ*^2^(3) = 11.5, *p* = 0.009). Older children selected the knowledgeable informant in significantly more trials than younger children (*p* = 0.001). There was no effect of the presence of screens (*p* = 0.786) or interaction between age and the presence of screens (*p* = 0.706). For full model output see Supplementary Table S2.

#### Alternative approaches in the pre-switch phase

**Figure 3:**
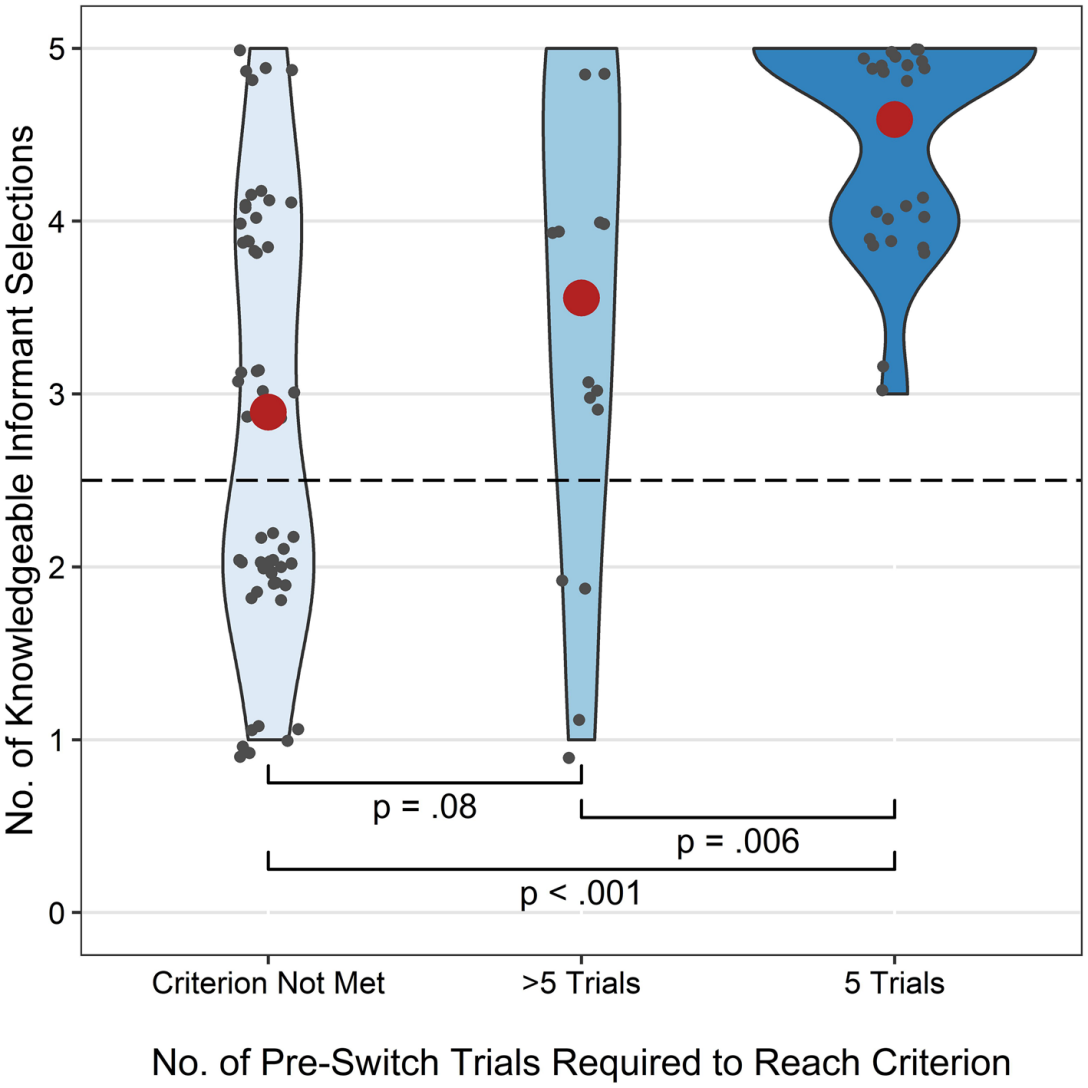
Number of switch trials in which children selected the knowledgeable informant by the number of pre-switch trials required to meet criterion. Children who met criterion after only five trials performed significantly better than children who took more than five trials to reach criterion and children who did not reach criterion. Small black points show individual participants’ performance, large red points indicate group means. Brackets indicate differences between groups. Dashed line indicates chance performance.

Of the 52 children who met criterion, 34 did so in only five pre-switch trials, while the rest required between six and 10 trials (Table 1). Meeting criterion after five pre-switch trials *and* continued success in the switch trials could indicate reasoning of the informants’ knowledge from task outset. Children who met criterion in five trials selected the knowledgeable informant more often in the switch trials than children who required more than five trials and children who did not reach criterion (Fig. 3). Full results given in Supplementary Information.

#### Categorising switch phase performance as explicitly metacognitive or implicit SLSs

Children who met criterion were categorised according to whether their behavioural response in the switch phase was considered likely to indicate employment of explicitly metacognitive or implicit SLSs. Children who selected the knowledgeable informant in at least four trials were considered likely to have reasoned about the informants’ perceptual access and were categorised as having employed an explicitly metacognitive SLS (*N* = 42). Children who selected the knowledgeable informant in fewer than four trials were categorised as likely having used an implicit SLS (*N* = 10). More older children fit the criteria for explicitly metacognitive SLSs than younger children (Table 2). Most children who used an implicit SLS required more than five trials to reach criterion. Children who did not meet criterion were not categorised as having employed either SLS.

**Table 2:** Categorising switch phase performance as explicitly metacognitive or implicit SLSs.

Age	*n*	Criterion met		Criterion not met
		Implicit SLSs	Explicitly metacognitive SLSs	
4 years	21	4 (0)	3 (2)	14 (0)
5 years	23	2 (0)	8 (4)	13 (1)
6 years	22	3 (0)	8 (6)	11 (3)
7 years	23	1 (0)	11 (11)	11 (6)
8 years	20	0 (0)	12 (10)	8 (2)

Children who selected the knowledgeable informant in at least four switch trials were categorised as having employed an explicitly metacognitive SLS. Children who did not meet criterion were not categorised as having employed either SLS. Numbers in parentheses indicate the number of children who provided a reasoned correct response to the explicit verbal reasoning question.

### Explicit verbal reasoning

Children’s responses to the explicit reasoning question were categorised: (0) no response; (1) non-reasoned responses (not related to the task, or comprised single words or gestures); (2) reasoned but incorrect responses (evidence of explicit reasoning, but motivations not related to visual perceptual access, or the answer was not sufficient to determine full correct reasoning) and (3) reasoned correct responses (clear reasoned evidence of explicit task understanding with reference to visual perceptual access). Reasoned correct responses were given by 41% of children.

**Figure 4:**
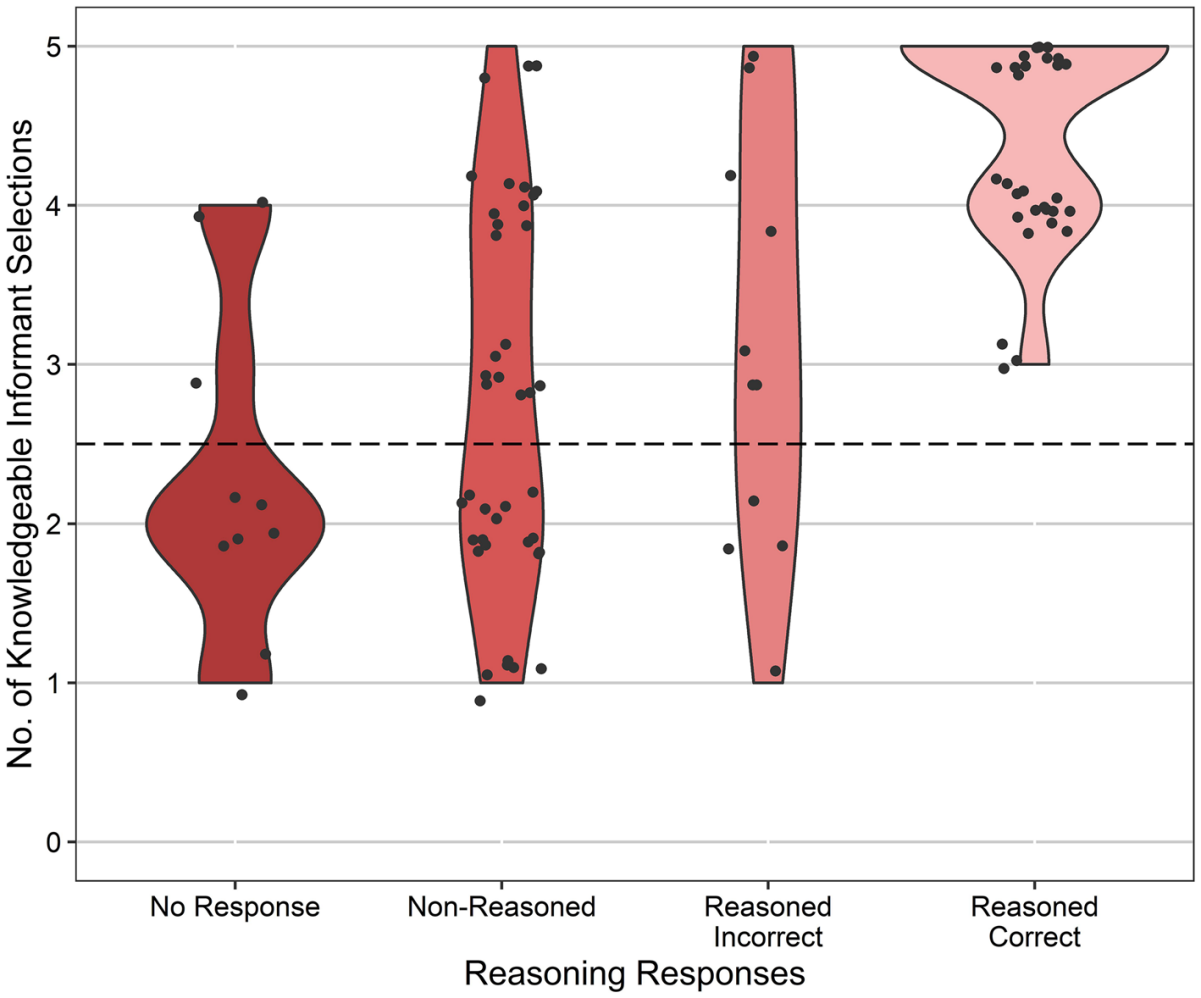
Number of knowledgeable informant selections by category of explicit verbal reasoning response. Children who were categorised as having provided a reasoned correct response were also more likely to have selected the knowledgeable informant in more switch trials. Points show individual participants’ performance. Dashed line indicates chance performance.

We investigated whether verbal reasoning was predicted by age, meeting the proficiency criterion, or switch trial performance. The results indicated that children who made more knowledgeable informant selections also provided better reasoned responses (Fig. 4). This effect of switch trial performance appeared to be greater in older children (Table 2). Full results given in Supplementary Information.

### Reasons for not reaching the proficiency criterion

We found no evidence to suggest that the informant selections of the 52% of children who did not reach criterion were influenced by any alternative yet consistent strategies (full results in Supplementary Information).

## Discussion

These results suggest that it is possible to differentiate implicit and explicitly metacognitive SLSs empirically. The methods we employed, drawn from existing paradigms in the comparative literature, facilitated investigation into the developmental trajectory of the transition from use of one process to the other. We showed that children who reached the proficiency criterion selected the knowledgeable informant in significantly more switch trials than children who did not reach criterion. This suggested that at least some children considered the informants’ perceptual access to identify which informant was knowledgeable. By contrast, the behaviour of children who did not reach criterion was not wholly consistent with either having formed an association, inferred a rule, or used an appropriate reasoning-based strategy, any of which could have led to success in the pre-switch phase. Accordingly, their informant selections in the switch trials were also less selective.

To investigate any age-related changes that indicated differences in the learning processes underlying children’s selections, we looked at the switch phase performance of those who met criterion. Here we found that older children sought information from a knowledgeable informant over an ignorant informant significantly more often than younger children. This developmental trend was consistent with our prediction that there would be age-related differences in the SLSs underpinning success in the pre-switch trials. We propose that, consistent with evidence from a previous study^19^, these results reveal a relatively late age-related transition from the use of implicit SLSs to the use of explicitly metacognitive SLSs. That older children in the present study selected the knowledgeable informant more often suggests that they likely had the capacity to assess others’ suitability as sources of knowledge based on their perceptual access (as inferred from personal experience). By contrast, at least some younger children appeared to have based their selections on something other than reasoning about the informants’ perceptual access, for example using visually salient, yet superficial, cues such as colour or side to learn a simple association or infer a rule (i.e., implicit SLSs). Our categorisation of particular patterns of behavioural responses is consistent with this interpretation. As the majority of learned associations or rules were no longer appropriate in the switch trials, this could explain why younger children made fewer selections of the knowledgeable informant despite reaching criterion.

Children who met criterion in only five pre-switch trials selected the knowledgeable informant significantly more often in the switch trials than both children who took more trials to reach criterion, and children who did not reach criterion. However, there was no difference between the latter two groups. These results suggest that the two groups of criterion-passers were employing different SLSs. Children who met criterion in five trials continued to select the knowledgeable informant in the switch trials, indicative of reasoning about the informants’ knowledge based on their perceptual access. Children who required more trials to reach criterion were relatively less successful, suggesting a greater reliance on implicit SLSs unrelated to the informants’ perceptual access. It is unlikely that all children in this group relied on implicit strategies, indeed the behavioural responses of some of this group fit the criteria for explicitly metacognitive SLSs, suggesting that they had reasoned about the informants’ perceptual access (albeit dependent on task experience to make this inference). Such disparity could explain why we see an average performance that is greater (though not significantly so) than children who did not reach criterion but lower than those who met criterion in five trials.

Removing the screens in the final two trials allowed us to explore whether participants were truly responding to the informants’ perceptual access, or if they had used a non-mentalistic cue to predict success. The lack of any effect of the presence of the screens on performance indicated that portraying perceptual access in a more visually salient manner did not make reasoning about informants’ perceptual access easier. It also suggested that children who continued to be successful in the switch trials were unlikely to have made selections based on an association, or rule, related to the colour of the frames. That is, children who recognised the value of perceptual access as a source of knowledge were able to use their personal experience of the screens to identify which of the informants had the desired knowledge, showing a similar capacity to human adults^68^.

Reasoning-based information seeking appears to be cognitively challenging, and its relatively late development indicates that the metacognitive capacities required may preclude younger children from identifying the most appropriate source of social information. As younger children appear to rely on implicit SLSs in place of explicitly reasoning about others’ mental states (necessary for success on this task), perhaps they have not yet developed the necessary metacognitive capacities to support explicitly metacognitive SLSs. Specifically, younger children might not yet have the capacity to recognise that perceptual access grants privileged knowledge, or if they do, may not appreciate that this renders an individual a valuable source of information.

It is not trivial that 52% of children did not reach the proficiency criterion, however, we were not expecting all children to do so, and in fact it was helpful to make comparisons against the pattern of informant selections made by this group. To get five consecutive correct trials, a successful rule needed to be inferred, or an association firmly established, by only the sixth trial. This was a high expectation for young children, and it is possible that in later trials some children did form an association, infer a successful rule, or even recognise the relevance of the informants’ perceptual access. Due to the nature of the methods used, we cannot be certain about the reasons for lower success of either the children who did not reach criterion, or those who struggled to switch from using implicit to explicitly metacognitive SLSs. They may have lacked the necessary conceptual understanding regarding the informants’ perceptual access or may not have been able to use such understanding to deduce which informant could provide valuable information. In previous studies^69^, younger children have struggled to recognise the potential value of others’ knowledge, despite reporting others’ knowledge based on their perceptual experience. The apparent cognitive challenge associated with actually using such information may have precluded many children from selecting the appropriate informant. These findings highlight just how challenging using explicit reasoning about others’ knowledge in the context of social learning is likely to be. Assumptions about the use of such strategies in the social learning literature should therefore be made and interpreted with extreme caution, especially with regards to the capacities of young children or animals.

Younger children’s failure to show the necessary flexibility of response during the switch trials could also have arisen for reasons unconnected to conceptual understanding. For instance, limited capacities for inhibition can lead to perseverative errors causing children to persist with a previously successful response despite knowledge of an updated rule^70,71^. However, we argue that children who recognised the significance of perceptual access should in fact give *this* rule primacy. Perseverating with a response other than one driven by the perceptual-access rule would suggest that they were likely attending to alternative cues. Developmental variation in comprehension of the questions used to prompt selection of an informant is also uncertain. These questions were comparable to “Ask” questions in the selective trust literature which are sometimes found to have a developmental trajectory^33,72^. However, some 4- and 5-year-olds in this study not only reached criterion, but were successful in the switch phase, and gave correctly reasoned verbal justifications. This suggests that success was not precluded by younger children's more limited capacities for comprehension.

Verbal justifications for informant selections offered an insight into children’s understanding of the benefits of perceptual access on others’ knowledge. We discovered that children who selected the knowledgeable informant more often in the switch trials were also more likely to have provided appropriate verbal reasoning for their choices. This is consistent with our interpretation that some children employed explicitly metacognitive SLSs based on a mentalistic causal understanding of the informants’ suitability as a source of information, though this effect was greater in older children. Given that explicit verbal reasoning responses were in line with our interpretation of the behavioural responses, the switch trials appeared to capture particular patterns of responses related to the use of implicit and explicitly metacognitive SLSs.

The developmental trend captured within our results may be indicative of a significant cognitive challenge associated with employing explicitly metacognitive SLSs. Hence, these metacognitive capacities may preclude younger children from recognising the value of others’ knowledge. Therefore, we argue that being able to fully benefit from others’ knowledge appears to be dependent, at least in part, on the ability to assess the others’ suitability as sources of knowledge based on their perceptual access to desired information. The relatively late development of explicitly metacognitive SLSs suggests that such capacities are unlikely to be observed in animals, offering credence to proposals that such capacities may be involved in distinctively human cumulative culture. If so, then the degree of flexibility afforded by an explicit understanding of others’ mental states, with regards to assessing others’ suitability as informants, may offer the significant advantage in social information use that drives human cultural evolution in a way not seen in animals.

## Method

### Participants

The final participant sample comprised 109 four- to eight-year-old children (55 females; *M* age = 76.8 months, *SD* = 16.9, range = 48–107). An additional 13 children were excluded from analyses due to: researcher error (*n* = 3), missing data including non-completion of the task (*n* = 7), and task interference (*n* = 3). Participants were visitors at RZSS Edinburgh Zoo, predominantly identified by parents/guardians as British. Most British children attend early learning and childcare facilities from around 3 years old, typically starting primary school between 4 and 5 years old. As in many WEIRD societies, British children’s early social experiences are generally facilitated by parents and other adult caregivers.

### Apparatus

Two adult females served as informants. To aid children in identifying the informants, each was referred to according to the pattern of their t-shirt (black with white polka dots, ‘*Spots*’, and black and white stripes, ‘*Stripes*’). All instructions were presented to informants and results recorded via a program written in PsychoPy v1.84.2^73^ run on a Microsoft Surface tablet. Two black wooden boxes (5.8 × 5.8 × 3.5 cm) served as potential reward locations; the target box contained a square Lego Duplo block, and the non-target box was empty. Two free-standing screens consisting of a yellow or blue wooden frame (35 × 35 cm) and black opaque or black semi-opaque inner screen (30 × 30 cm) were used to manipulate informants’ perceptual access (Fig. 1; based on materials used by Karg and colleagues^74^). The semi-opaque screen with a blue frame permitted perceptual access when viewed at a close distance (experience phase; Fig. 1a), though when viewed at an angle or from a distance it appeared opaque (pre-switch and switch phases; Fig. 1b). The opaque screen with a yellow frame prevented perceptual access. A larger opaque screen (40 × 30 cm) with a yellow frame (45 × 35 cm) occluded the participant’s view. A differently coloured laminated card was fixed to each side of the testing table.

### Procedure

**Figure 5:**
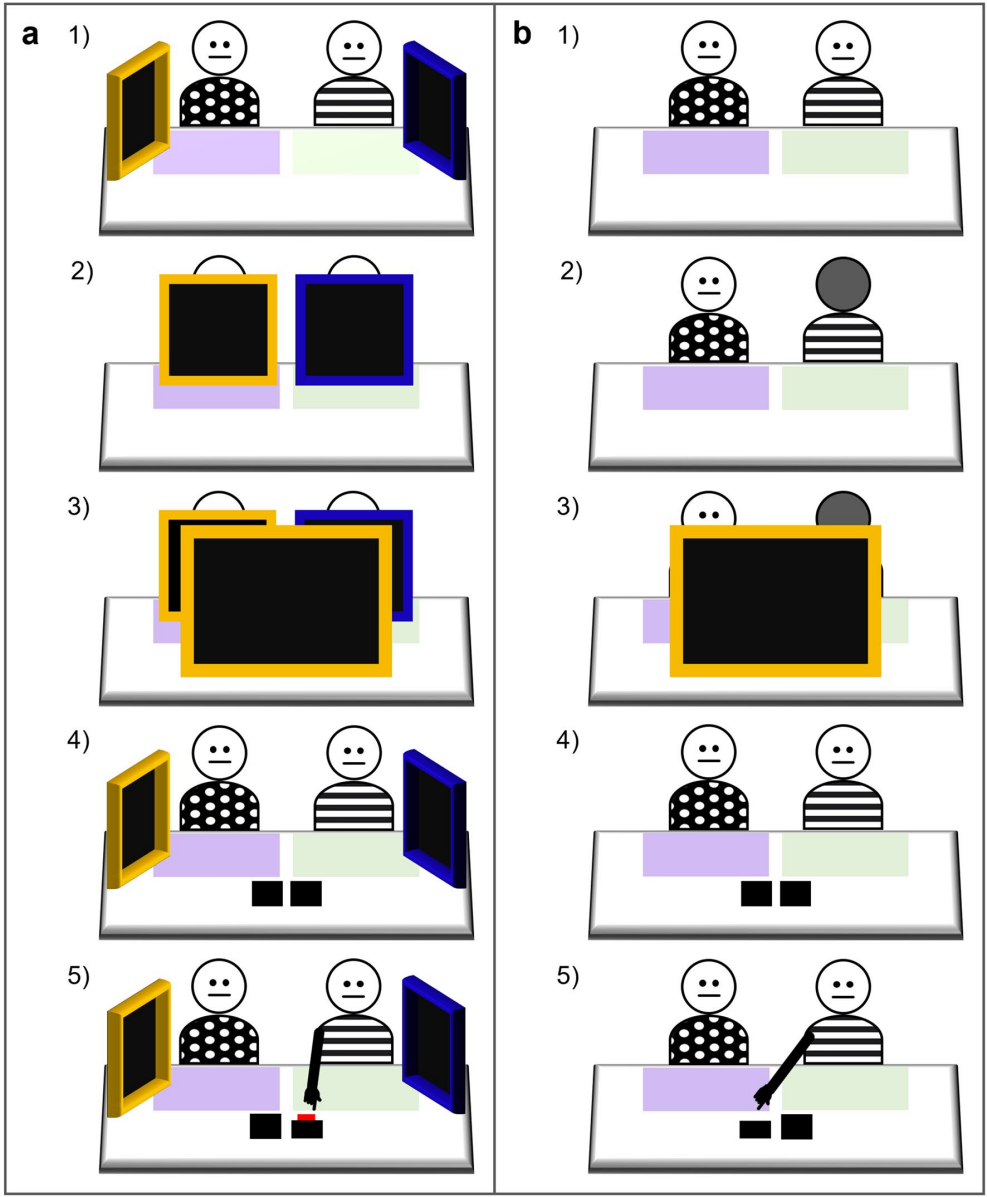
Schematic illustration from the perspective of the participant, including placement of frames and boxes. (**a**) example of trials in which perceptual access is indicated by opaque (yellow) and semi-opaque (blue) screens, as in all pre-switch trials and the first three switch trials; (**b**) example of trials in which perceptual access is indicated by the informants facing or not facing the critical event (i.e., the ignorant informant faced away), as in the final two switch trials. The stages illustrated are: (**1**) start of the trial, (**2a**) screens placed in front of informants, (**b**) ignorant informant turns away, (**3**) opaque (yellow) screen occludes participant’s view, behind the screen two boxes (one containing Lego) are mixed around and closed, (**4a**) screens removed, (**b**) ignorant informant turns back around, (**5**) participant selects informant, reward revealed or not revealed depending on phase and selection.

Participants took part individually in a single testing session for which they received a sticker. The two informants sat next to each other, across the table from the experimenter and the participant (Fig. 5a(1)).

#### Experience phase

Participants were introduced to the two types of screens and shown that they could see through the blue semi-opaque screen, but not through the yellow opaque screen (Fig. 1a). To check participants’ understanding of their own and others’ perceptual access, children were asked which screen they could see the experimenter through, and which screen the experimenter could see them through. Errors were corrected by the experimenter before moving on to the pre-switch phase (*n* = 4).

#### Pre-switch phase

The experimenter introduced the informants Spots and Stripes. Participants were then shown two boxes, one that contained Lego (target) and one that was empty (non-target). The experimenter explained that they were going to try and find the Lego to build a tower. Before hiding the Lego, the semi-opaque (blue) screen was placed in front of one informant and the opaque (yellow) screen was placed in front of the other (Fig. 5a(2)), this rendered the informants’ knowledgeable and ignorant respectively. From the participant’s position both informants’ screens appeared to be opaque (Fig. 1b). The larger opaque (yellow) screen was then placed in front of the participant to occlude their view of the table and the informants’ screens (Fig. 5a(3)). In the centre of the table, between the participant’s and informants’ screens, the experimenter mixed around the open boxes before closing them to hide the Lego. The participant’s screen was removed, and the informants moved their respective screens to the sides of the table (Fig. 5a(4)). The experimenter asked the participant “*Who do you think will tell you where the Lego is hidden?*” Regardless of which informant was chosen, the knowledgeable informant opened the target box to reveal the Lego (i.e., these trials were ‘no-risk’ as the reward was always revealed; Fig. 5a(5)). The participant could then retrieve the Lego and start building a tower. The same procedure was repeated for up to 10 pre-switch trials, beginning with showing the contents of the boxes. The starting position of the knowledgeable informant (semi-opaque screen) was randomly generated on the first pre-switch trial. The semi-opaque and opaque screens then remained in the same positions throughout the pre-switch trials, meaning that the position of the knowledgeable informant was also constant. The sides on which Spots and Stripes sat were pseudo-randomly determined for each trial, such that if the participant completed all 10 pre-switch trials there would be an equal distribution of trials in which Spots and Stripes were on each side. Thus, although the position of the knowledgeable informant was consistent, the identity of the informant (Spots or Stripes) was not. Therefore, both informants were epistemically competent, with the situation (position of the screens) dictating their knowledge on a given trial.

Participants progressed to the switch phase of the task either after they had selected the knowledgeable informant in five consecutive pre-switch trials (met criterion), or after 10 pre-switch trials. To reach criterion participants could have explicitly reasoned the informants’ potential to provide valuable information based on their perceptual access to the critical event (when the Lego was being hidden), or they could have used superficial, yet salient, visual cues to form an association, or infer a rule, that predicted success. Appropriate associations or rules could have been based on the colour of the side of the table, the side of the table, or the colour of the frame. Any one of these cues may have been more salient to participants than perceptual access, and in this phase would have had the same desired outcome. Not all children were expected to reach criterion due to the limited number of trials in which to form an association, or infer a rule, that predicted success.

#### Switch phase

This phase began with a ‘switch’ in the task scenario. The switch referred to the random switching of the positions of the knowledgeable and ignorant informants in each trial. The five switch trials followed a similar format to the pre-switch trials, with two key procedural changes.

First, the positions of the knowledgeable informant (semi-opaque screen) no longer remained constant. Switching the position of the screens rendered most learned associations, or inferred rules, ineffective (except for frame colour). In the first trial the semi-opaque and opaque screens switched positions to the opposite of their pre-switch phase positions. In the following four trials, the side of the semi-opaque screen (knowledgeable informant) was randomly assigned, thus sometimes switching and sometimes not.

In the first three switch trials, the informants’ perceptual access continued to be indicated by the two screen types. However, in the final two trials perceptual access was indicated by the knowledgeable informant facing, and the ignorant informant facing away from, the critical event (see Fig. 5b for an example of a trial). These ‘no screens’ trials were included to explore whether participants were truly responding to the informants’ perceptual access, or whether they had simply made the connection between the reward and the screen, or frame colour, that they could have continued to use as a predictive cue. Consistent success across the different types of switch trial would strongly suggest that participants were explicitly reasoning about the informants’ perceptual access to the critical event and that they understood why their behaviour was informative.

The second key change related to the selection that participants were asked to make. Instead of being asked who they thought would tell them where the Lego was hidden, the participant was asked “*Who do you ****want**** to tell you where they think the Lego is hidden?*” The change to this question was also reflected in the informants’ reactions to participants’ selections. In contrast to the pre-switch phase, the informant chosen by the participant selected a box and opened it to reveal its contents; the knowledgeable informant opened the target box, while the ignorant informant opened the non-target box (illustrated in Fig. 5b).

### Ethical approval

Ethical approval was granted by the University of Stirling General University Ethics Panel (GUEP673). Informed written consent was obtained via the child’s parent/guardian. Research was conducted in accordance with relevant guidelines and regulations.

### Informed consent

Informed written consent was obtained via the child’s parent/guardian. Research was conducted in accordance with relevant guidelines and regulations.

## Supplementary Information

Supplementary Information.

## Data Availability

Data and analysis code are available at https://osf.io/et6ub/.
