# Supplementary material for: Children transition from simple associations to explicitly reasoned social learning strategies between age four and eight
Source: Sci Rep. 2022 Mar 23;12:5045. doi: 10.1038/s41598-022-09092-1 (PMC8943005; doi:10.1038/s41598-022-09092-1)
Supplement: Supplementary file 1 — Supplementary Information. [file 41598_2022_9092_MOESM1_ESM.pdf]

Supplementary information

Children transition from simple associations to explicitly reasoned social learning strategies between age four and eight

Kirsten H. Blakey, Elizabeth Renner, Mark Atkinson, Eva Rafetseder, and Christine A. Caldwell

Results

Switch phase performance

Effect of reaching the proficiency criterion

Table S1. Effect of reaching the proficiency criterion on switch trial performance.

	b	SE	*Z*	*p*
age	0.99	0.27	3.61	< .001
criterion met	0.75	0.14	5.46	< .001
screens	−0.08	0.13	−0.61	.544
age:criterion met	0.58	0.27	2.14	.033
age:screens	0.03	0.24	0.14	.886
criterion met:screens	0.03	0.12	0.24	.808
age:criterion met:screens	0.11	0.24	0.48	.634

Age-effects in children who met the proficiency criterion

Table S2. Age-effect of reaching the proficiency criterion on switch trial performance.

	b	SE	*Z*	*p*
age	1.63	0.51	3.18	.001
screens	−0.05	0.20	−0.27	.786
age:screens	0.16	0.42	0.38	.706

Alternative approaches in the pre-switch phase

A GLMM was built for selection of the knowledgeable informant in each switch trial with fixed effects of age, whether five or more pre-switch trials were required to reach criterion, the presence of screens, and all the interactions between these variables, with a random intercept of

participant ID. This model was significantly better than the null equivalent ($\chi^2(7) = 25.6$, $p < .001$). A significant main effect of age ($p = .004$) indicated that older children selected the knowledgeable informant more often than younger children. A significant main effect of the number of trials required to meet criterion ($p = .002$) suggested that children who met criterion after five trials selected the knowledgeable informant more often than children who required more trials. Again, there was no evidence of an effect of the presence of screens ($p = .648$) or any interactions between the fixed effects ($p \geq .074$). For the full model output see Table S3.

Table S3. Effect of the number of pre-switch trials required to reach criterion on switch trial performance.

	b	SE	*Z*	*p*
age	1.45	0.50	2.88	.004
pre-switch trials	0.80	0.26	3.10	.002
screens	−0.11	0.24	−0.46	.648
age:pre-switch trials	−0.02	0.50	−0.04	.968
age:screens	0.14	0.47	0.30	.767
pre-switch trials:screens	−0.43	0.24	−1.79	.074
age:pre-switch trials:screens	−0.32	0.47	−0.68	.489

One-sample *t*-tests revealed that children who met criterion in five trials selected the knowledgeable informant significantly above chance, in an average of 4.59 switch trials $t(33) = 20.0$, $p < .001$, two-tailed. Children who required more trials to reach criterion also selected the knowledgeable informant significantly more often than chance in an average of 3.56 trials $t(17) = 3.24$, $p = .005$, two-tailed. A one-way ANOVA comparing the performance of these groups to children who did not reach criterion revealed a significant difference between the three groups $F(2, 106) = 24.15$, $p < .001$ (Fig. 3). Post hoc Tukey HSD tests showed that children who met criterion in five trials ($M = 4.59$ trials, $SD = 0.61$, $N = 34$) selected the knowledgeable informant significantly more often than both children who required more trials ($M = 3.56$ trials, $SD = 1.38$, $N = 18$, $p = .006$) and children who did not reach criterion ($M = 2.89$ trials, $SD = 1.26$, $N = 57$; $p < .001$). The difference between the latter two groups was not significant ($p = .08$).

Explicit verbal reasoning

We investigated whether verbal reasoning was predicted by age, meeting the proficiency criterion, or switch trial performance by conducting ordinal regressions (using the *ordinal* package[53] in R). Reasoning responses were submitted to an ordinal regression with fixed effects of age, meeting criterion, and the interaction between these variables. Significant main effects of age ($p < .001$) and meeting criterion ($p < .001$; Fig. S1) suggested that older children and children who met criterion provided better reasoned responses than younger children and children who did not meet criterion, respectively. There was no evidence of a significant interaction between age and meeting criterion ($p = .305$). For the full model output see Table S4.

Table S4. Effect of age and meeting criterion on explicit verbal reasoning response.

	b	SE	*Z*	*p*
age	1.98	0.44	4.50	< .001
criterion met	0.83	0.21	3.97	< .001
age:criterion met	0.83	0.42	1.03	.305

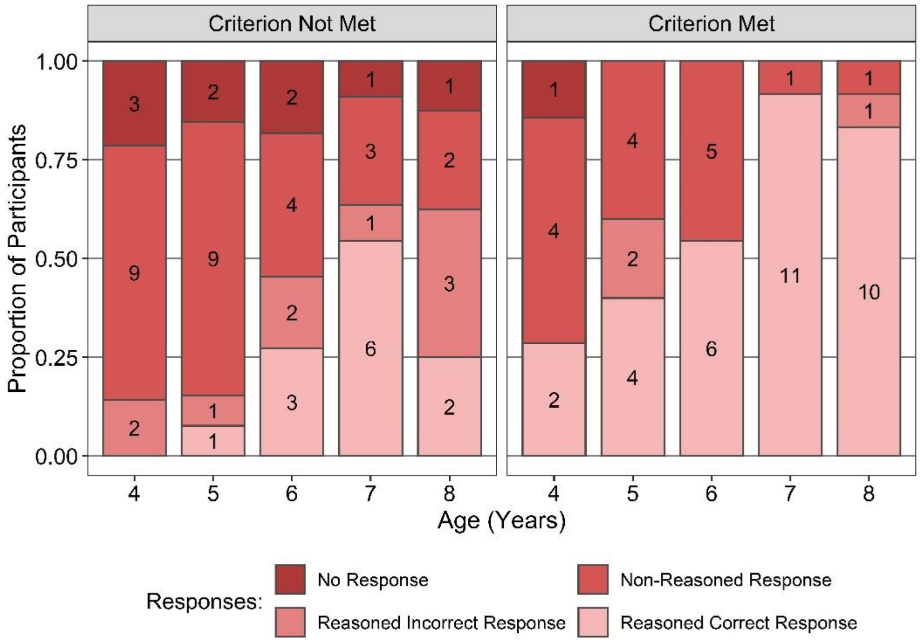

Figure S1. Proportion of participants providing each category of explicit verbal reasoning responses by age in years and whether children met criterion. Values presented on bars refer to the number of participants each proportion represents.

A further ordinal regression explored the relationship between switch trial performance and verbal reasoning. This model included fixed effects of age, meeting criterion, switch trial performance, and the interactions between these variables. A significant main effect of switch trial performance ($p < .001$) indicated that children who made more knowledgeable informant selections also provided better reasoned responses (Fig. 4). There were no significant main effects of age (p = .287) or meeting criterion (p = .133). However, a significant interaction between switch trial performance and age (p = .032) was likely, at least in part, the product of older children's greater switch trial performance. For full model output see Table S5.The effect of switch trial performance appeared to be enhanced by age. That is, given the absence of a main effect of age, this interaction suggests that older children who performed well in the switch trials were more likely to provide a better reasoned response than younger children who also performed well (Table 2).

Table S5. Effect of switch trial performance, age, and meeting criterion on explicit verbal reasoning response.

	b	SE	*Z*	*p*
performance	1.30	0.26	5.00	< .001
age	−1.93	1.81	−1.07	.287
criterion met	−1.42	0.95	−1.50	.133
performance:age	1.01	0.47	2.14	.032
performance:criterion met	0.44	0.24	1.83	.068
age:criterion met	−0.81	1.80	−0.45	.651
performance:age:criterion met	−0.01	0.47	−0.02	.982

Reasons for not reaching the proficiency criterion

We also explored the possibility that the 52% of children who did not meet criterion were employing alternative yet consistent strategies regarding informant selections. There were a number of potential strategies that could have interfered with children's formation of an association, or inference of a rule, in the number of trials available to them. For example, children may have preferred a particular informant, they may have alternated their choices between the sides, or they may not have used any strategy at all. A chi-square test of independence showed no evidence of an overall preference for either informant (p = .675) or of any particular age group (years) having a preference for either informant (p = .717). To explore whether children had any preferences for copying or not copying the side they selected on the previous trial we conducted further chi-square

tests of independence. These revealed no evidence of preference for either copying or not copying the side selected on the previous trial (p = .895) and no evidence of that any age group preferred one of these choices (p = .533). These results suggest that it is unlikely that children who did not reach criterion were using a consistent alternative strategy for informant selections.

Of the 34 children who did not reach criterion, 12 provided reasoned correct verbal justifications suggesting that had there been more trials in which to reach criterion they may have done so. One child's justification appeared to be drawn from the final two trials without screens (*"at the end I knew it could be stripes because spots was turned away and couldn't see it"*). Nine children gave reasoned but incorrect verbal justifications. The strategies indicated by these children included references to patterns and turn taking (e.g., *"I found a pattern, it switched from Spots to Stripes from Spots to Stripes", "because I was taking turns"*).
